# Effects of Pesticides on Occupationally Exposed Humans

**DOI:** 10.1100/tsw.2006.207

**Published:** 2006-09-25

**Authors:** Stylianos M. Piperakis, Konstantina Kontogianni, Michael M. Piperakis, Ricardo Marcos, Smaragdi Tsilimigaki

**Affiliations:** ^1^ Biology Unit, Department of Pre-School, Education University of Thessaly, Volos 38221, Greece; ^2^ DNA Repair Laboratory, Institute of Biology, National Center for Scientific Research “Demokritos”, 153 10 Aghia Paraskevi, Athens, Greece; ^3^ Department of Chemistry, University of Sheffield, United Kingdom; ^4^ Grup de Mutagenesi, Departament de Genetica I de Microbiologia, Universitat Autonoma de Barcelona, 08193 Bellaterra, Spain

**Keywords:** comet assay, DNA damage, DNA repair, greenhouses, pesticides

## Abstract

Pesticides are known to contain numerous genotoxic compounds; however, genotoxicity biomonitoring studies of workers occupationally exposed to pesticides have produced variable results. In this study, we employed the Comet assay to examine DNA damage in peripheral blood lymphocytes (PBLs) from 64 greenhouse workers from Almería in south-eastern Spain in comparison to PBLs from 50 men from the same area but not engaged in any agricultural work. The results indicated that there were no differences in the basal levels of DNA damage in the two study groups. In addition, exposure of PBL from the workers and controls to hydrogen peroxide or γ-irradiation led to similar levels of DNA damage; the subsequent repair of the induced DNA damage was also similar for both study populations. Smoking had no impact on any of the responses. The results of this study indicate that the greenhouse workers had no detectable increase in DNA damage or alteration in the cellular response to DNA damage compared to our control population.

### INTRODUCTION

Farmers are occupationally exposed to mixtures of pesticides. Beside acute injuries, long-term effects are suspected to result from these exposures. Over 1,000 chemicals have been classified as pesticides, while a number of them are potentially genotoxic[1,2,3,4]. Some studies have shown significantly increased risks of lung cancer[5], bladder cancer[6,7], and leukemia[8,9,10] in workers exposed to pesticides.

Among the many *in vitro* and *in vivo* assays that have been used to evaluate the induction of DNA damage and mutation by pesticides, the lymphocyte micronucleus assay perhaps has been the most widely used[11,12,13,14]. Genotoxic compounds have been found among all the major types of pesticides, including fungicides, insecticides, and herbicides. A number of epidemiological studies also have been conducted to ascertain genetic risk, at least at the somatic cell level, for human populations[6,14,15,16,17,18].

Several studies have reported an association between occupational exposure to pesticides and increased levels of chromosome aberrations and/or sister chromatid exchange (SCE) in peripheral blood lymphocytes (PBLs)[19,20]. The single cell gel electrophoresis (SCGE) assay, or comet assay (CA), also has been used in biomonitoring studies of pesticide-exposed workers[13,21,22]. This assay has been used for a number of different types of toxicology studies, ranging from mechanistic studies and clinical investigations to biomonitoring and molecular epidemiology investigations[23].

In the present study the CA was used to compare long-term DNA damage in PBL from 64 pesticide-exposed greenhouse workers from Almería in southeastern Spain and in PBL from a nonexposed reference group living in the same area and with similar general characteristics. The CA data were also analyzed in relation to smoking habits to determine whether this potential confounding factor influenced levels of DNA damage. Finally, experiments were conducted to measure the levels of DNA damage induced by hydrogen peroxide and γ-irradiation in PBLs from worker and control populations, and the extent of repair of that damage, in order to investigate whether occupational exposure results in DNA repair efficiency alterations.

### MATERIALS AND METHODS

#### Chemicals and Media

Plastics were from Corning (Corning, NY). RPMI 1640 medium with phenol red, fetal calf serum, phytohemagglutinin (PHA), and trypan blue were obtained from Biochrom KG (Berlin, Germany). Lymphoprep was supplied by Nycomed (Oslo, Norway). L-Glutamine, penicillin, and streptomycin were from ICN Flow (Irvine, CA). Low and normal melting point agarose were supplied by Gibco BRL (U.K.). Phosphate-buffered saline tablets (PBS), dextrose, hydrogen peroxide ($H_2O_2$), and 4′,6-diamidine-2-phenylindole dihydrochloride (DAPI) were obtained from Sigma (St. Louis, MO).

#### Study Population

A group of 64 male agricultural workers from the province of Almería (Spain), occupationally exposed to different mixtures of pesticides, was recruited for this study. In the group, 73% of the individuals worked on ornament plants, 17% on vegetables, and 10% on both. Table 1 gives the main pesticides used in this region, classified as fungicides, bactericides, and insecticides, with an indication of their frequency of use.

The control group consisted of 50 healthy men from the same area, without previous occupational exposure to pesticides (civil servants). Table 2 presents the main demographic characteristics and exposure information (i.e., age, smoking habits, years of pesticide exposure for both groups). They differ with respect to their average age, but this can be easily accommodated in the statistical analysis by introducing the age as a covariate.

Prior to the study, each individual provided informed consent. Blood samples were collected and further manipulated in accordance with the ethical standards. All volunteers were healthy individuals living in rural environment. At the time of drawing blood samples, a personal history questionnaire was completed. The questionnaire covered standard demographic questions (age, living area, etc.) as well as medical (genetic disorders, number of X-rays diagnoses, vaccinations, medications, etc.), lifestyle (smoking, diet, etc.), and occupational questions (working hours/day, years of exposure, etc.). For the exposed group, a further questionnaire was completed including specific questions related to farming: kind of crops, pesticide application, use of protective measures, etc.. The questionnaires used were similar to that published by the International Commission for Protection against Environmental Mutagens and Carcinogens[24].

**TABLE 1**
**Pesticides used for spraying by the studied group, with indication of their frequencies of use, WHO classification by hazard, and mutagenicity (M) and carcinogenicity (C) experimental data**

| Type | Product | Use | Class (WHO) | M | C |
|---|---|---|---|---|---|
| Fungicides | Carbendazim | 3.1 | * | + | - |
| | Cymoxanil | 14.1 | * | * | - |
| | Diethofencarb | 3.1 | * | * | * |
| | Mancozeb | 12.5 | IV | + | - |
| | Nuarimol | 3.1 | * | + | * |
| | Fosetyl-aluminium | 6.2 | V | - | - |
| | Procymidone | 10.9 | V | - | - |
| | Propamocarb | 3.1 | * | - | * |
| | Propineb | 7.8 | V | - | - |
| Bactericides | Kasugamycin | 4.7 | V | - | - |

| Type | Product | Use (%) | Class (WHO) | M | C |
|---|---|---|---|---|---|
| Insecticides | Abamectine | 35.9 | * | - | - |
| | Acrinathrin | 17.2 | * | * | * |
| | Buprofezin | 4.7 | IV | - | + |
| | Cyromazine | 12.5 | IV | * | - |
| | Dichlorvos | 3.2 | Ib | + | + |
| | Endosulfan | 20.3 | II | + | - |
| | Formetanate | 9.4 | I | - | - |
| | Imidacloprid | 50 | * | + | - |
| | Malathion | 12.5 | III | + | - |
| | Methamidophos | 34.4 | Ib | + | - |
| | Methomyl | 50 | II | + | - |
| | Oxamyl | 14.1 | Ib | - | - |
| | Permethrin | 4.7 | II | - | - |
| | Pyriproxyfen | 14.1 | * | * | * |
| | Tebufenozide | 4.7 | * | * | * |
| | Tralomethrin | 15.6 | * | * | * |

*, Not available: -, no observed effects; +, adverse effects in at least one experiment (See M.L.Richardson, 1992, for more detailed information).

**TABLE 2**
**Characteristics of the Studied Groups**

| | Control | Exposed | t |
|---|---|---|---|
| No. subjects | 50 | 64 | |
| Age (years)[a] | 38.56 ± 1.35 | 32.83 ± 1.13 | 3.235* |
| Years of exposure | — | 9.82 ± 1.04 | |
| Smoking habits | | | |
| No. nonsmokers | 20 | 29 | |
| No. smokers | 30 | 35 | |
| Cigarettes/day[a] | 14.70 ± 1.57 | 12.54 ± 1.65 | 0.927 |

[a]Mean ± S.E.; *$p = 0.001$.

### Lymphocyte Isolation, Cryopreservation, and Thawing

A 5-ml blood sample was obtained from each volunteer by venipuncture using heparinized vacutainers with diluted 1:1 with RPMI 1640 (pH 7.3) and kept on ice for 15 min. The blood was layered onto 5-ml Lymphoprep and the PBLs were isolated by centrifugation at 200 × *g* for 30 min. The buffy coats were removed and washed twice with RPMI 1640. PBLs suspended in RPMI were counted in a hemocytometer and then cryopreserved. For the cryopreservation, the cell suspension was centrifuged at 200 × *g* for 5 min, and the cell pellet was resuspended at a concentration of $10 \times 10^6$ cells/ml in a freezing medium consisting of 10% dimethylsulfoxide, 40% RPMI 1640, and 50% fetal calf serum. Aliquots of $2 \times 10^6$ cell suspension were transferred to plastic freezing vials. The vials were placed in a Cryo 1°C freezing container, then directly in a –70°C freezer so as to achieve a –1°C/min cooling rate; frozen cell stocks were stored at –70°C[25].

Vials were retrieved as needed and submerged in a 37°C water bath until the last trace of ice was melted. The thawed PBLs were quickly transferred to conical centrifuge tubes containing 15 ml of prechilled thawing medium consisting of 50% fetal calf serum, 40% RPMI, and 10% dextrose (one tube/vial). The cells were centrifuged at 200 × *g* for 10 min at 4°C and the cell pellet was resuspended in ice-cold PBS (pH 7.3) for use in the CA. Cell viability, using trypan blue, was found to be over 95% for both untreated and treated cells[26].

### Hydrogen Peroxide ($H_2O_2$) and γ-Irradiation Treatments and Repair Studies

Thawed PBL were suspended in RPMI 1640 medium and placed in microcentrifuge tubes at a concentration of $1 \times 10^5$ cells/tube. The cells then were exposed to 150 µ*M* $H_2O_2$ on ice for 5 min or 4.2 Gy of γ-irradiation from a $^{60}$Co source.

To examine repair of the DNA damage caused by exposure of the PBL to $H_2O_2$ or γ-irradiation, the cells were resuspended in 1-ml RPMI 1640 medium supplemented with 20% fetal calf serum, 2 m*M* L-glutamine, and penicillin, and transferred into a well of a 24-well plate (Corning). PHA was added to the wells to produce a final concentration of 2.4 µg/ml, and the plates were incubated for 2 h at 37°C in a humidified atmosphere of 5% $CO_2$. Further incubation of up to 24 h did not increase the extent of repair measured after 2 h[26].

### Single Cell Gel Electrophoresis

The SCGE assay was performed under alkaline conditions using an adaptation of previously described methods[27,28,29,30]. Cells with or without $H_2O_2$ and γ-irradiation treatment were suspended in molten 1% low melting point agarose in PBS (pH 7.4) at 37°C, and 100 µl were spread onto a microscope slide precoated with 100 µl of 1% agarose. The agarose was solidified on ice for 10 min, followed by immersion of the slides in a lysis solution (2.5 *M* NaCl, 100 m*M* $Na_2$EDTA, 10 m*M* Tris, NaOH to pH 10.0, and 1% Triton-X-100) for 1 h at 4°C, in order to remove cellular proteins. The slides then were placed in an electrophoresis tank containing 0.3 *M* NaOH and 1 m*M* $Na_2$EDTA (pH >13) for 40 min. Subsequently, electrophoresis was performed using the same buffer at 25 V (1 V/cm, 300 mA) for 30 min at an ambient temperature of 4°C. The slides then were washed three times for 5 min each, with 0.4 *M* Tris-HCl (pH 7.5), at 4°C before staining with 5 µg/ml DAPI[31,32].

### Evaluation of DNA Damage

DAPI-stained nucleoids were examined at 400× magnification with a WANG epi-fluorescence microscope (WANG BioMedical, Amsterdam, The Netherlands), equipped with a 350-nm excitation filter and a 420-nm barrier filter. One hundred comets on each slide were scored visually as belonging to one of five predefined classes according to tail intensity and were given a value of 0, 1, 2, 3, or 4 (from undamaged, 0, to maximally damaged, 4). Thus, the total score for 100 comets could range from 0 (all undamaged) to 400 (all maximally damaged) in arbitrary units[22]. To confirm the visually scored result, the percentage of DNA in comet tails was estimated using an image analysis system (Kinetic Analysis, Wirral, U.K.) connected to a computer with a suitable program.

### Statistical Analysis

For each donor, 300 comets per treatment condition (100 comets/slide, triplicate slides/treatment) were used to evaluate DNA damage and repair. Mean scores, in arbitrary units (±SD), were calculated from the respective values, as well from the image analysis estimates. A multivariate analysis of variance (GLM-Repeated Measures) was used to evaluate differences in the distribution of DNA damage and repair in the group of 64 male agricultural workers and the 50 healthy men who served as controls. A level of at least 0.05 was used to determine significance. To detect differences between groups with regard to the mean value of confounding factors (age, smoking, etc.), the Mann-Whitney U-test was applied due to the observed departure from normality.

## RESULTS

Fig. 1 presents DNA migration data for PBL from the worker and control populations stratified by their smoking habits (basal damage). DNA damage was measured by visual scoring of the comets. DNA damage was also measured following a 5-min treatment with 150 μ*M* $H_2O_2$. Analysis of variance indicated that there were no significant differences in the level of basal DNA damage between the nonsmoker controls and the nonsmoker greenhouse workers ($p > 0.05$) and also between smoker controls and smoker greenhouse workers ($p > 0.05$). Although the smoker groups had somewhat increased levels of DNA damage, the observations indicated that neither smoking nor exposure to pesticides had a significant effect on the basal levels of DNA damage.

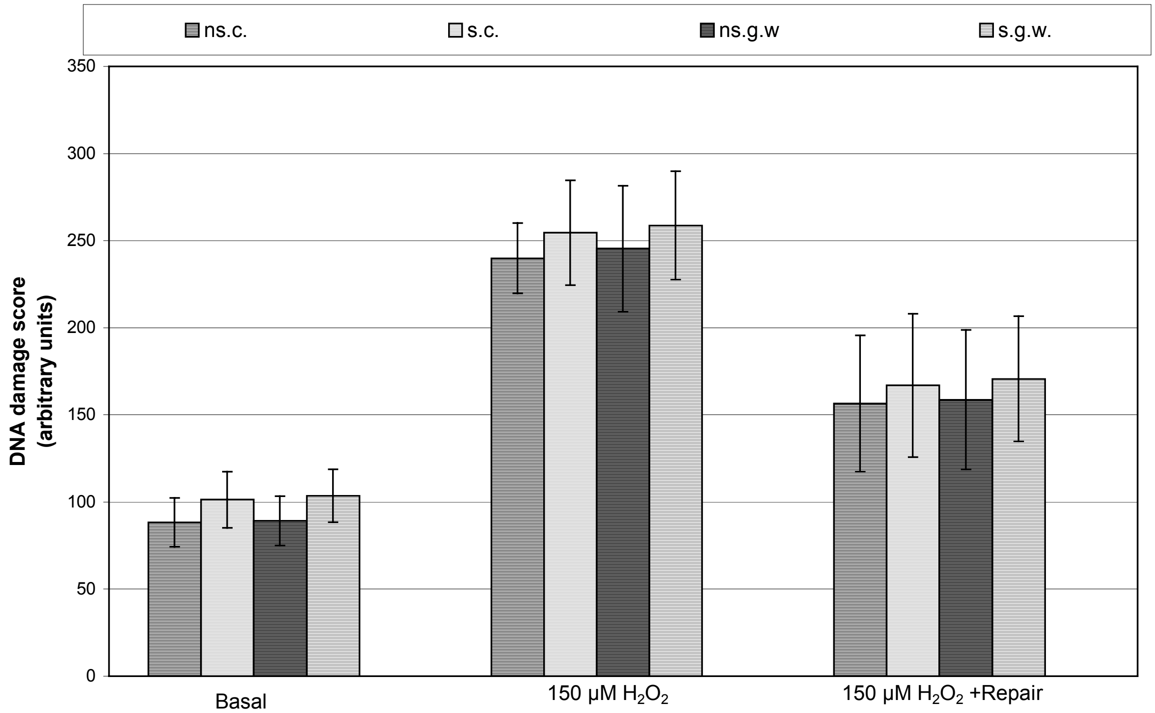

**FIGURE 1.** Response of lymphocytes of the groups [nonsmokers control (ns.c); smokers control (s.c.); nonsmokers greenhouse workers (ns.g.w.); smokers greenhouse workers (s.g.w.)] after exposure to 150 μ*M* $H_2O_2$ and after repair. Error bars represent the standard deviation of the mean among the donors.

Treatment with 150 μ*M* $H_2O_2$ produced a significant increase in DNA damage in comparison to the level of DNA damage in the corresponding untreated group ($p < 0.05$). This increase in DNA damage, however, was similar for the workers and controls and was not influenced by smoking, indicating that the working conditions had no effect on the oxidative DNA damage induced by the $H_2O_2$ treatment.

Similar results were found when lymphocytes from greenhouse workers and controls were treated with 4.2 Gy of γ-rays (Fig. 2). The treatment resulted in a significant increase in DNA damage compared with the level of DNA damage detected in the corresponding untreated PBL. The increase in DNA damage was similar for the worker and control PBLs, and was not affected by prior exposure to tobacco smoke.

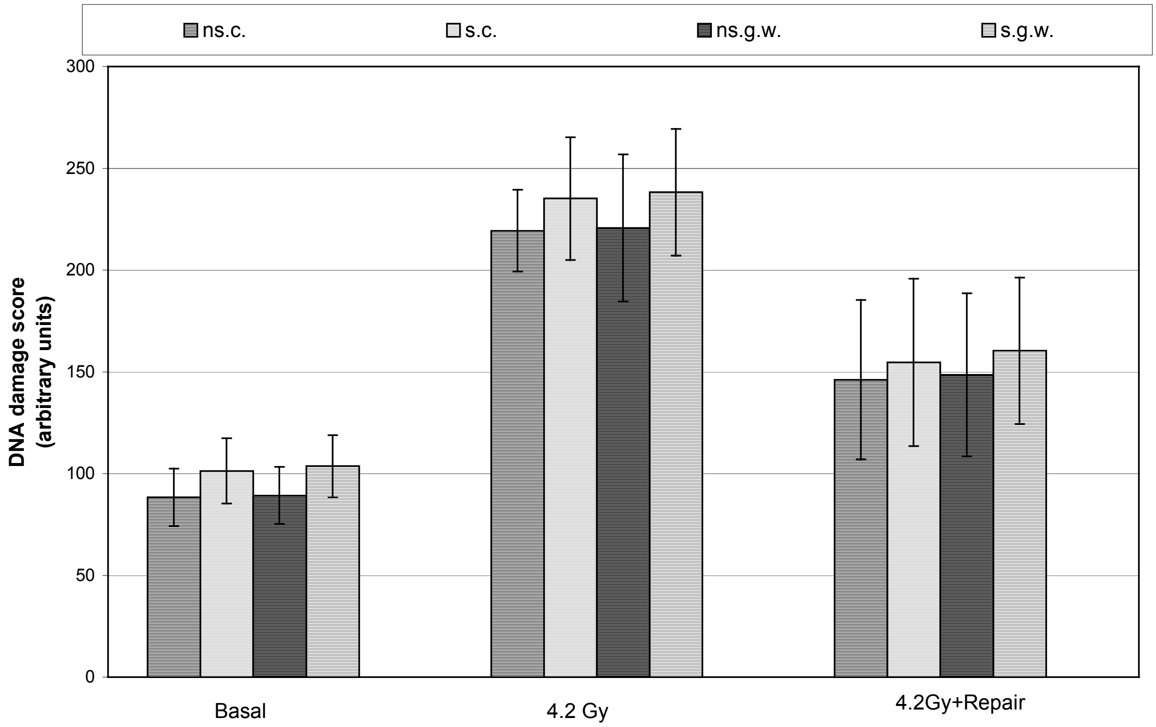

**FIGURE 2.** Response of lymphocytes of the groups [nonsmokers control (ns.c); smokers control (s.c.); nonsmokers greenhouse workers (ns.g.w.); smokers greenhouse workers (s.g.w.)] after exposure to 4.2 Gy $^{60}Co$ and after repair. Error bars represent the standard deviation of the mean among the donors.

A 2-h repair period resulted in a significant ($p < 0.05$) decrease in the initial level of damage produced by the $H_2O_2$ treatment. The degree of repair of the $H_2O_2$-induced damage was similar for PBLs from greenhouse workers and the controls, and was unaffected by smoking (Fig. 1). Repair had a similar effect on the DNA damage induced by γ-rays; the 2-h incubation resulted a significant ($p < 0.05$) decrease in the initial level of damage, and the degree of the decrease was similar for smoker and nonsmoker workers and controls.

DNA damage was also measured by the percent DNA in the comet tail using the Kinetic Analysis image analysis system (Table 3). The results using this metric of DNA damage were similar to those obtained by microscopic classification of comets (Figs. 1 and 2).

**TABLE 3**
**Mean Values of DNA Damage as Measured by Image Analysis System in the Four Groups**

| | Mean ± SD | | | |
|---|---|---|---|---|
| **Samples** | **Men, Nonsmokers Control** | **Men, Smokers Control** | **Men, Nonsmokers Pesticides** | **Men, Smokers Pesticides** |
| Control | 16.73 ± 5.3 | 19.30 ± 5.9 | 16.91 ± 7.8 | 19.76 ± 3.5 |
| 150 μ*M* $H_2O_2$ | 46.76 ± 4.4 | 49.69 ± 2.3 | 47.87 ± 2.7 | 50.50 ± 3.3 |
| 150 μ*M* H2O2 + Repair | 30.23 ± 3.1 | 32.31 ± 2.8 | 30.68 ± 2.9 | 33.06 ± 2.1 |
| 4.2 Gy $^{60}$Co | 42.69 ± 1.1 | 45.82 ± 1.9 | 42.97 ± 3.1 | 46.46 ± 3.1 |
| 4.2 Gy $^{60}$Co + Repair | 28.18 ± 2.1 | 29.87 ± 3.5 | 28.66 ± 1.6 | 31.02 ± 3.7 |

[a]As measured by image system analysis ± SD.

### DISCUSSION

By definition, pesticides are toxicants intended to control pest populations. Although the benefits associated with their use in agriculture are unquestionable, many of their active substances have potentially adverse effects on human health. A number of cytogenetic biomonitoring studies have been performed on agricultural workers from different regions who were subjected to a variety of exposure conditions. Yoder et al.[33] noted a marked increase in chromatid lesions in lymphocyte cultures prepared from individuals exposed to pesticides during heavy spraying periods. Carbonell et al.[34,35] found that the frequency of chromosome aberrations is related to the degree of the pesticides exposure. Crossen et al.[36] and Dulout et al.[37,38] found significantly elevated frequency of SCE in lymphocytes from subjects occupationally exposed to pesticides. Kourakis et al.[39,40] also observed increased clastogenicity in human somatic cells from workers occupationally exposed to pesticides. However, several similar studies did not detect increased DNA damage in pesticide-exposed workers[41,42,43,44]. Several biomonitoring studies found an increase in micronucleus frequency in workers with occupational exposure to pesticides[18,45,46], although negative results also have been reported[14,17,44]. Finally, conflicting results have been found using the CA[13,21,22]. Some of the variability in the results of these studies may be explained by exposure of the various study populations to different pesticides, and by the use of assays having different sensitivities and specificities[44].

Cigarette smoking is a well-documented source of a variety of potentially mutagenic and carcinogenic compounds. Smoking, however, has produced conflicting results in human biomonitoring studies that measured micronuclei and DNA damage using the CA[47,48,49,50]. $H_2O_2$ readily penetrates the cell membrane and initiates the generation of highly reactive species through the transition metal-catalyzed Haber-Weiss reaction[51,52]. In order to produce DNA damage, a concentration of $H_2O_2$ must be present that is sufficient to overwhelm the cellular antioxidant capacity. Antioxidant defences include antioxidant enzymes (catalase), scavenger molecules, and the ability of cells to remove altered molecules by turnover[53]. γ-Irradiation is able to break DNA directly by deposition of energy in the deoxyribosephosphate backbone; single-strand breaks and double-strand breaks are produced. However, most of the energy is deposited in water leading to hydroxyl radicals that can subsequently react with bases and sugars in DNA producing base modifications, sites of base loss (abasic sites), and strand breaks[26].

The agricultural workers were selected for the present study because of the particular characteristics of their working area; the agricultural activity is intensive and exclusively in greenhouses, suggesting the potential for a high level of pesticide exposure. In addition, the climatic conditions of the area allow 3–4 crops/year, which implies a constant use and application of pesticides throughout the year. The results of our study were in agreement with the results of a previous study carried in the same population using micronuclei as the endpoint[14]. This previous study also failed to find significant differences between the pesticide-exposed population and control populations. Taken together, the results of these studies indicate that this specific group of agricultural workers, under their particular exposure conditions, do not have significantly increased levels of DNA damage. Our results also indicate that PBLs from these workers are damaged by $H_2O_2$ and γ-irradiation similarly to PBLs from controls, and that the repair of the DNA damage produced by $H_2O_2$ and γ-irradiation is similar to that of the controls. As far as smoking habits are concerned, our CA data indicated that the smokers had slightly more DNA damage than the nonsmokers; however, this difference was not significant.

### ACKNOWLEDGMENTS

This work was supported by EU grant no.ERBICISCT960300 to S.M. Piperakis. We thank Dr. G. Efstathiou of the University of Athens, Greece for helping us in the statistical analysis.

## References

1. Tezuka, H., Ando, N., Suzuki, R., Terahata, M., Moriya, M., and Shirasu, Y. (1980) Sister-chromatid exchanges and chromosomal aberrations in cultured Chinese hamster cells treated with pesticides positive in microbial reversion assays. *Mutat. Res*. **78**, 184–191.
2. Waters, M.D., Sandhu, S.S., Simmon, V.F., Mortelmans, K.E., Mitchell, A.D., Jorgenson, T.A., Jones, D.L., Valencia, R., and Garret, N.E. (1982) Study of pesticide genotoxicity. In *Genetic Toxicology*. Fleck, R.A. and Hollaender, A., Eds. Plenum, New York. pp. 279–326.
3. Garret, N.E., Stack, H.F., and Waters, M.D. (1986) Evaluation of the genetic activity profiles of 65 pesticides. *Mutat. Res*. **168**, 301–325.
4. Sinha, S.P. (1989) Genotoxicity of pesticides. *Perspect. Cytol. Genet*. **6**, 749–753.
5. Barthel, E. (1981) Increased risk of lung cancer in pesticide-exposed male agricultural workers. *J. Toxicol. Environ. Health* **8**, 1027–1040.
6. De Ferrari, M., Arfuso, M., Bonassi, S., Bonatti, S., Cavalieri, Z., Pescatore, D., Marchini, E., Pisano, V., and Abbondandolo, A. (1991) Cytogenetic biomonitoring of an Italian population exposed to pesticides: chromosome aberration and sister-chromatid exchange analysis in peripheral blood lymphocytes. *Mutat. Res*. **260**, 105–113.
7. Viel, J.F. and Chalier, B. (1995) Bladder cancer among French farmers: does exposure to pesticides in vineyards play a part? *Occup. Environ. Med*. **52**, 587–592.
8. Brown, L.M., Blair, A., Gibson, R., Everett, G.D., Cantor, K.P., Schuman, L.M., Burmeister, L.F., van Lier, S.F., and Dick, F. (1990) Pesticide exposures and other agricultural risk factors for leukemia among men in Iowa and Minnesota. *Cancer Res*. **50**, 6585–6591.
9. Blair, A. and Zahm, S.H. (1995) Agricultural exposures and cancer. *Environ. Health Perspect.* **103**, 205–208.
10. Blair, A., Zahm, S.H, Pearce, N.E., Heineman, E.F., and Fraumeni, J. (1992) Clues to cancer etiology from studies of farmers. *Scand. J. Work Environ. Health* **18**, 209 –215.
11. Bianchi, L., Zannoli, A., Pizzala, R., Stivala, L.A., and Chiesara, E. (1994) Genotoxicity assay of five pesticides and their mixtures in *Saccharomyces cerevisiae* D7. *Mutat. Res*. **321**, 203–211.
12. Bernacchi, F., Ponzanelli, I., Barale, R., and Loprieno, N. (1996) Mutagenic activity of some coal-derived humic compounds evaluated by the Ames test. *Mutat. Res*. **369**, 107–112.
13. Garaj-Vrhovac, V. and Zeljezic, D. (2000) Evaluation of DNA damage in workers occupationally exposed to pesticides using single-cell gel electrophoresis (SCGE) assay. Pesticide genotoxicity revealed by comet assay. *Mutat. Res*. **469**, 279–285.
14. Lucero, L., Pastor, S., Suárez, S., Durbán, R., Gómez, C., Parrón, T., Creus, A., and Marcos, R. (2000) Cytogenetic biomonitoring of Spanish greenhouse workers exposed to pesticides: micronuclei analysis in peripheral blood lymphocytes and buccal epithelial cells. *Mutat. Res.* **464**, 255–262.
15. Pastor, S., Creus, A., Xamena, N., Siffel, C., and Marcos, R. (2002) Occupational exposure to pesticides and cytogenetic damage: results of a Hungarian population study using the micronucleus assay in lymphocytes and buccal cells. *Environ. Mol. Mutagen*. **40**, 101–109.
16. Pastor, S., Gutierrez, S., Creus, A., Xamena, N., Piperakis, S., and Marcos, R. (2001) Cytogenetic analysis of Greek farmers using the micronucleus assay in peripheral lymphocytes and buccal cells. *Mutagenesis* **16**, 539–545.
17. Pastor, S., Creus, A., Parron, T., Cebulska-Wasilewska, A., Siffel, C., Piperakis, S., and Marcos, R. (2003) Biomonitoring of four European populations occupationally exposed to pesticides: use of micronuclei as biomarkers.

*Mutagenesis* **18**, 249–258.
18. Bolognesi, C., Landini, E., Perrone, E., and Roggieri, P. (2004) Cytogenetic biomonitoring of a floriculturist population in Italy: micronucleus analysis by fluorescence in situ hybridization (FISH) with an all-chromosome centromeric probe. *Mutat. Res*. **557**, 109–117.
19. Rita, P., Reddy, P.P., Venkatram, S., and Reddy, O.S. (1987) Monitoring of workers occupationally exposed to pesticides in grape gardens of Andhra Pradesh. *Environ. Res*. **44**, 1–5.
20. Rupa, D.S., Reddy, P.P., and Reddy, O.S. (1989) Analysis of sister-chromatid exchanges, cell kinetics and mitotic index in lymphocytes of smoking pesticide sprayers. *Mutat. Res*. **223**, 253–258.
21. Lebailly, P., Vigreux, C., Lechevrel, C., Ledemeney, D., Godard, T., Sichel, F., Le Talaer, J.Y., Henry-Amar, M., and Gauduchon, P. (1998) DNA damage in mononuclear leukocytes of farmers measured using the alkaline comet assay: discussion of critical parameters and evaluation of seasonal variations in relation to pesticide exposure. *Cancer Epidemiol. Biomark. Prev.* **10**, 917–927.
22. Piperakis, S.M., Petrakou, E., Tsilimigaki, S., Sagnou, M., Monogiudis, E., Haniotakis, G., Karkaseli, H., and Sarikaki, E. (2003) Biomonitoring with the comet assay of Greek greenhouse workers exposed to pesticides. *Environ. Mol. Mutagen*. **41**, 104–110.
23. Collins, A.R. (2002) The comet assay. Principles, applications and limitations. *Methods Mol. Biol.* **203**, 163–177.
24. Carrano, A.V. and Natarajan, A.T. (1988) Considerations for population monitoring using cytogenetic techniques. ICPEMC Publication No. 14. *Mutat. Res*. **204**, 379–406.
25. Dimitroglou, E., Zafiropoulou, M., Messini-Nikolaki, N., Doudounakis, S., Tsilimigaki, S., and Piperakis, S.M. (2003) DNA damage in human population affected by chronic psychogenic stress. *Int. J. Hyg. Environ. Health* **206**, 39–44.
26. Visvardis, E.E., Tassiou, A.M., and Piperakis, S.M. (1997) Study of DNA damage induction and repair capacity of fresh and cryopreserved lymphocytes exposed to $H_2O_2$ and γ-irradiation with the alkaline comet assay. *Mutat. Res*. **383**, 71–80.
27. Collins, A.R., Ma, A.G., and Duthie, S.J. (1995) The kinetics of repair of oxidative DNA damage (strand breaks and oxidised pyrimidines) in human cells. *Mutat. Res*. **336**, 69–77.
28. Singh, N.P., Stephens, R.E., Singh, H., and Lai, H. (1999) Visual quantification of DNA double-strand breaks in bacteria. *Mutat. Res*. **429**, 159–168.
29. Piperakis, S.M., Visvardis, E.-E., and Tassiou, A.M. (1999) Comet assay for nuclear DNA damage. *Methods Enzymol*. **300**, 184–194.
30. Tsilimigaki, S., Messini-Nikolaki, N., Kanariou, M., and Piperakis, S.M. (2003) A study on the effects of seasonal solar radiation on exposed populations. *Mutagenesis* **18**, 139–143.
31. Piperakis, S.M., Petrakou, E., and Tsilimigaki, S. (2000) Effects of air pollution and smoking on DNA damage of humans lymphocytes. *Environ. Mol. Mutagen*. **36**, 243–249.
32. Tronov, V.A., Konstantinov, E.M., Petrakou, E., Tsilimigaki, S., and Piperakis, S.M. (2002) Nicotinamide "protects" resting lymphocytes exposed to hydrogen peroxide from necrosis but not from apoptosis. *Cell Biol. Toxicol*. **18**, 359–367.
33. Yoder, J., Watson, M., and Benson, W. (1973) Lymphocyte chromosome analysis of agricultural workers during extensive occupational exposure to pesticides. *Mutat. Res*. **21**, 335–340.
34. Carbonell, E., Xamena, N., Creus, A., and Marcos, R. (1993) Cytogenetic biomonitoring in a Spanish group of agricultural workers exposed to pesticides. *Mutagenesis* **8**, 511–517.
35. Carbonell, E., Valbuena, A., Xamena, N., Creus, A., and Marcos, R. (1995) Temporary variations in chromosomal aberrations in a group of agricultural workers exposed to pesticides. *Mutat. Res*. **344**, 127–134.
36. Crossen, P.E., Morgan, W.F., Horan, J.J., and Stewart, J. (1978) Cytogenetic studies of pesticide and herbicide sprayers. *N. Z. Med. J*. **88**, 192–195.
37. Dulout, F.N., Pastori, M.C., Olivero, O.A., Gonzalez, Cid M., Loria, D., Matos, F., Sobel, U., De Bujan, E.C., and Albiano, N. (1985) Sister-chromatid exchanges and chromosomal aberrations in a population exposed to pesticides. *Mutat. Res*. **143**, 237–244.
38. Dulout, F.N., Lopez Camelo, J.S., and Guradze, H.N. (1992) Analysis of sister chromatid exchanges in human population studies. *Rev. Braz. Genet*. **15**, 169–182.
39. Kourakis, A., Mouratidou, M., Kokkinos, G., Barbouti, A., Kotsis, A., Mourelatos, D., and Dozi-Vassiliades, J. (1992) Frequencies of chromosomal aberrations in pesticide sprayers working in plastic green houses. *Mutat. Res*. **279**, 145–148.
40. Kourakis, A., Mouratidou, M., Barbouti, A., and Dimikiotou, M. (1996) Cytogenetic effects of occupational exposure in the peripheral blood lymphocytes of pesticide sprayers. *Carcinogenesis* **17**, 99–101.
41. Hogstedt, B., Kolnig, A.M., Mitelman, F., and Skerfving, S. (1980) Cytogenetic studies of pesticides in agricultural work. *Hereditas* **92**, 177–178.
42. De Cassia, S., Becak, R.W., Gaeta, R., and Rabello-Gay, M.N. (1982) Cytogenetic study of workers exposed to methyl-parathion. *Mutat. Res*. **103**, 71–76.
43. Hoyos, L.S., Carvajal, S., Solano, L., Rodriguez, J., and Orozco, L. (1996) Exposed to pesticides in Colombia. *Environ. Health Perspect*. **104(Suppl. 3),** 535–538.
44. Scarpato, R., Migliore, L., Angotai, G., Fedi, A., Migli, L., and Loprieno, N. (1996) Cytogenetic monitoring of a group of Italian floriculturists, no evidence of DNA damage related to pesticide exposure. *Mutat. Res.* **367**, 73–82.
45. Bolognesi, C., Parrini, M., Bonassi, S., Ianello, G., and Salanitto, A. (1993) Cytogenetic analysis of a human population occupationally exposed to pesticides. *Mutat. Res*. **285**, 239–249.
46. Joksic, G., Vidakovic, A., and Spasojevic-Tisma, V. (1997) Cytogenetic monitoring of pesticide sprayers. *Environ. Res*. **75**, 113–118.
47. Au, W.W., Cajas-Salazar, N., and Salama, S. (1998). Factors contributing to discrepancies in population monitoring studies. *Mutat. Res*. **400**, 467–478.
48. Bonassi, S., Neri, M., Lando, C., Ceppi, M., Lin, Y.P., Chang, W.P., Holland, N., Kirsch-Volters, M., Zeiger, E., and Fenech, M. (2003). Effect of smoking habit on the frequency of micronuclei in human lymphocytes: results from the Human MicroNucleus project. *Mutat. Res.* **543**, 155–166.
49. Hininger, I., Chollat-Namy, A., Sauvaigo, S., Osman, M., Faure, H., Cadet, J., Favier, A., and Roussel, A.M. (2004) Assessment of DNA damage by comet assay on frozen total blood: method and evaluation in smokers and non-smokers. *Mutat. Res*. **558**, 75–80.
50. Hoffmann, H. and Speit, G. (2005) Assessment of DNA damage in peripheral blood of heavy smokers with the comet assay and the micronucleus test. *Mutat. Res*. **581**, 105–114.
51. Meneghini, R. (1988) Genotoxicity of active oxygen species in mammalian cells. *Mutat. Res.* **195**, 215–230.
52. Ames, B.N. (1989) Endogenous oxidative DNA damage, aging and cancer. *Free Radic. Res. Commun.* **7**, 121–128.
53. Remacle, J., Raes, M., Toussaint, O., Renard, P., and Rao, G. (1995) Low levels of reactive oxygen species as modulators of cell function. *Mutat. Res*. **316**, 103–122.
